# Northwest Texas Medical Society; Austin District Medical Society, Seventeenth Quarterly Meeting

**Published:** 1891-11

**Authors:** 


### Society Notes.

The Northwest Texas Medical Association will hold its regular semi-annual meeting at Cisco, on the first Tuesday in December, convening at 3 p. m. The following papers will be read and discussed:

Pneumonia—Dr. Vance; discussion opened by Dr. Florence.

Puerperal Fever—Dr. Harrington; discussion opened by Dr. Isbell.

Fracture of Thigh—Dr. W. M. Powell; discussion opened by Dr. Rodman.

Placenta Prævia—Dr. Salmon; discussion opened by Dr. Stout.

New Therapeutical Remedies—Dr. R. G. Powell; discussion opened by Dr. Pipkin.

If not already a member, attend this meeting, and we guarantee you will be amply repaid for your time.

P. C. Coleman, Secretary.

C. F. Paine, President.

---

Seventeenth Quarterly Meeting of the Austin District Medical Society, and joint session with the Central Texas Medical Association, at Austin, Texas, Thursday, December 17, 1891:

Dear Doctor:—It affords us pleasure to announce that the Central Texas Medical Association will meet with us in joint session at this, our seventeenth quarterly meeting, and participate in the programme here subjoined. The Central Texas Association meets quarterly, at Waco, and is one of the largest and most influential medical organizations in the State, and numbers amongst its members some of the ablest men in the profession.

It need hardly be said that an unusually interesting and profitable time is anticipated. We will have a large attendance, and you are cordially invited to be present; and if you are not already a member, join us, and take part in the proceedings.

You will observe that this is our annual meeting, at which officers for the ensuing year are to be elected, and the usual banquet given, Bear in mind, also, that the payment of annual dues will be in order.

The Society will meet in Medical Hall, 513 Congress avenue, and be called to order promptly at 10 a. m.

J. W. McLAUGHLIN, President.

T. J. BENNETT, Secretary.

PROGRAMME—MORNING SESSION, 10 O'CLOCK.

1. Address of welcome.
2. Reading of minutes.
3. Applications for membership.
4. "Enteritis Simulating Bowel Obstruction," by Dr. Daniel Parker, of Calvert; discussion opened by Dr. J. M. Frazier, of Waco, and Dr. W. A. Howard, of Waco, all of the C. T. M. A.
5. "Perityphlitis," by Dr. W. R. Blailock, of McGregor; discussion opened by Dr. G. B. Foscue, of Waco, and Dr. W. E. Brown, of Gatesville, all of the C. T. M. A.

AFTERNOON SESSION, 3 O'CLOCK.

6. "Is Syphilis Transmitted Directly from the Father to the Child?" Affirmative: Dr. C. O. Weller, of Austin; Negative: Dr. W. T. Richmond, of Manor, both of the A. D. M. S.
7. "The Management of Abortions," by Dr. J. W. Carhart, of Lampasas; discussion opened by Dr. T. D. Wooten, of Austin, and Dr. G. W. Christian, of Burnet, all of the A. D. M. S.
8. Voluntary papers.
9. Verbal reports of cases.
10. Unfinished business.
11. New business.
12. Election of officers.
13. Retiring President's address.
14. Banquet at night.

---

The Treatment of Occipito—Posterior Position.—Dr. A. Worcester, in the *Journal of the American Medical Association*, July 4th, calls attention to a modification of the forceps methods of rotating the occiput to the anterior position. He suggests that the hand be introduced into the uterus, and used as a guide to the blades in adjusting them to the child's head. When the blades are introduced and applied without thus knowing how they are fitted to the child's head, the delivery is rarely easy or safe.

